# Rerouting of the tract in the treatment of high anal fistula: a single-center experience

**DOI:** 10.1007/s10151-025-03179-3

**Published:** 2025-07-24

**Authors:** Mahmoud Refaat Shehata, Mahmoud Mohamed Mohamed Abdelghany, Gamal Abdel-Hamid Ahmed Eid, Ragai Sobhi Hanna

**Affiliations:** Surgery Department, Faculty of Medicine, Assiut University, Assiut, Egypt

**Keywords:** Rerouting, Tract, Treatment, High anal fistula

## Abstract

**Background:**

High anal fistulae require more complicated treatment than low anal fistulae. Because of their complexity, this study aimed to assess the rerouting role in high anal fistulae treatment, as well as to assess recurrence and incontinence, and determine whether rerouting of the tract is a good option for treating high anal fistulae.

**Methods:**

This is a prospective interventional study that was conducted on 83 patients with high perianal fistula, ranging in age from 18 to 72 years old, of both genders. All cases were assigned to history taking, laboratory investigations, clinical examination (general examination and local examination), and magnetic resonance imaging (MRI) for objective delineation of the fistulous tract and its association to the anal sphincters.

**Results:**

- After a minimum follow-up period of 9 months, five cases (6.02%) experienced recurrence. Mild incontinence was reported in four patients (4.8%), while four patients (4.8%) developed infection. In addition, tract gangrene was observed in two patients (2.41%).
- In multivariate regression analysis, suprasphincteric fistulae and infection were independent predictors for recurrence.

**Conclusions:**

The rerouting procedure is a feasible and safe surgical option for managing high transsphincteric perianal fistulae. It is associated with low postoperative complication rates, including short-term recurrence. It combines the advantages of fistulotomy and sphincter-preserving fistula surgery. However, further studies involving a large number of suprasphincteric fistula cases are needed to evaluate the efficacy of the rerouting technique in treating such fistulae.

## Introduction

High anal fistulae represent a complex form of perianal disease, often involving deep structures and significant challenges in diagnosis and treatment. The distinction between low and high anal fistulae is critical in guiding both surgical management and the prediction of outcomes [1]. Various classifications have been proposed to classify these fistulae as low or high; simple or complex; or intersphincteric, transsphincteric, suprasphincteric, or extrasphincteric on the basis of their anatomy [2]. High fistulae tend to have more intricate anatomy and are associated with higher rates of recurrence and complications, requiring specialized care [1].

**Fig. 1:**
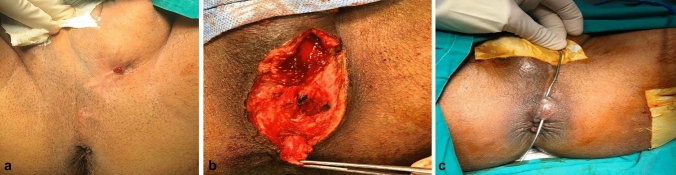
**A** External opening before surgical intervention. **B** Interposition of the track in the intersphincteric groove. **C** The second stage, laying open the intersphincteric track

The primary treatment for anal fistula is surgical intervention. The optimal treatment strategy is to eliminate the infected lesion, facilitate the closure of the fistula, and ensure adequate drainage while avoiding the risk of injury to the anal sphincters [3] (Fig. 1).

The actual hazard associated with these fistulae is the potential for incontinence in the case that a significant section of the external sphincter is inadvertently severed or injured [4].

Rerouting is a transposition technique that was introduced by Mann and Clifton in 1985 for the treatment of high anal and anorectal fistulae [5]. The procedure entails the immediate restoration of the external sphincter and the rerouting of the extra sphincteric portion of the tract into an intersphincteric position. The external sphincter is effectively recovered at a later date, and the newly positioned intersphincteric fistula is eventually addressed [6].

**Fig. 2:**
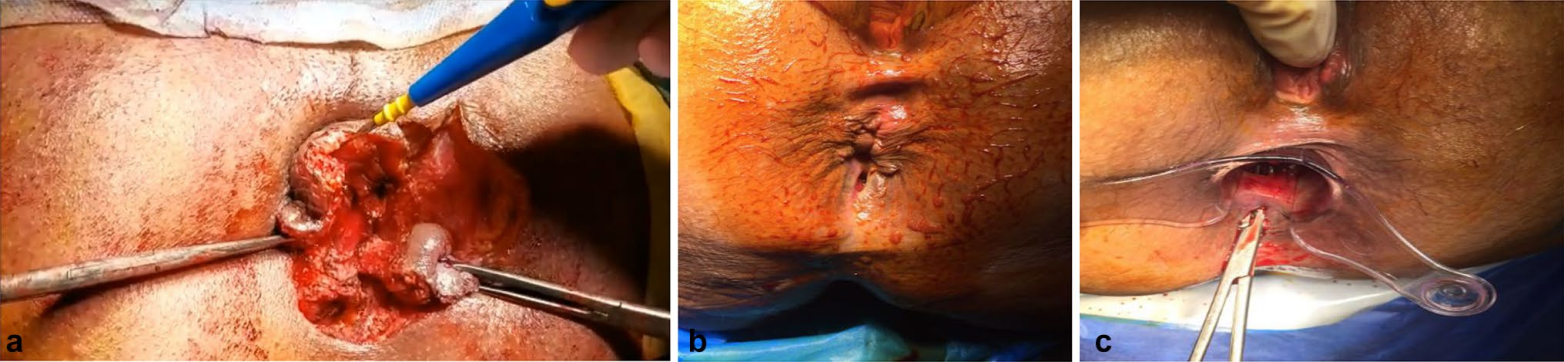
**A** Dissection of the tract up to its entrance through the external sphincter. **B** Second stage, preoperative. **C** Lay open fistulotomy

This study’s purpose is to evaluate the role of rerouting the tract in high anal fistulae treatment, as well as to assess recurrence and incontinence, and determine if rerouting of the tract is a good choice in the treatment of high anal fistula (Fig. 2).

## Patients and methods

This study is the first to be based on a large number of high anal fistula cases. It is a prospective interventional study that was carried out on 83 patients of both sexes with high perianal fistula, aged 18–72 years old, from September 2021 to January 2024 with follow up to September 2024 (Table 1).

**Table 1:** The Jorge–Wexner incontinence score

Type of incontinence	Frequency				
	Never	Rarely	Sometimes	Usually	Always
Solid	0	1	2	3	4
Liquid	0	1	2	3	4
Gas	0	1	2	3	4
Wears pad	0	1	2	3	4
Lifestyle alteration	0	1	2	3	4

Never = 0; rarely =  < 1/month; sometimes =  < 1/week but > 1/month; usually =  < 1/day but > 1/week; always =  > 1/day

### Ethical approval

This study was approved by the Assiut Faculty of Medicine Institutional Review Board (approval number: 17101821) at Assiut University, Egypt, and was conducted in the general surgery department at Assiut University Hospitals. Written informed consent was obtained from all patients included in the study. The study was registered with ClinicalTrials.gov (registration number: NCT05476146) on 27 July 2022, and was conducted in accordance with the Declaration of Helsinki.

### Study inclusion and exclusion criteria

This study included all patients with high cryptoglandular anal fistulae, whether de novo or recurrent.

The exclusion criteria were as follows:

- Cases involving low anal fistulae, defined as those where the fistula tract is located in the lower third of the external anal sphincter.
- Patients with fistulae secondary to other pathologies, such as malignancy, inflammatory bowel disease, trauma, or radiation.
- Patients who exhibited incontinence during the preoperative evaluation were also excluded.

### Data collection

Each case was examined for the following: history-taking (personal history, current complaint, analysis of each complaint, review of other gastrointestinal tract [GIT] symptoms, review of other systems, current medical comorbidities, and previous surgical history) and clinical examination (general examination and local examination, entire perineum, digital rectal examination, association between the anorectal ring and the tract position before the cases received anesthesia and anoscopy). Laboratory investigations and magnetic resonance imaging fistulogram were required for all cases: for the objective description of the fistulous tract and its association with the anal sphincters.

### Preoperative preparation

The night prior to the operation, patients were advised to undergo a brief mechanical bowel preparation that involved a single rectal enema and a restriction of oral consumption to eliminate fluids 12 h prior to the operation.

### The surgical technique

All patients underwent the two-stage rerouting operation under spinal anesthesia when the patient was in the lithotomy position. The site of the operation was properly prepared and draped. Before the skin incision, a broad-spectrum antibiotic was commenced (intravenous [IV] ciprofloxacin 400 mg) in addition to IV metronidazole (500 mg).

### The first stage

Coring out of the fistulous tract was done via cutting and coagulation diathermy. The surgical dissection was stopped at the point where the tract traversed the external anal sphincter. Following this, a circumanal incision was made at the anal verge, centered on the point where the fistulous tract pierced the external sphincter. The intersphincteric space was entered and dissected until we felt the fistulous tract. The fistulous tract was dissected from the external sphincter by simple muscle splitting, and it was pulled into the intersphincteric space. The opening in the external sphincter was obliterated by a few interrupted stitches using absorbable suture material (Vicryl 2/0 sutures). If the tract was too long, its distal portion was excised. No seton was inserted in the fistulous tract.

### The second stage

This was carried out 6–8 weeks after the first stage. The second stage was also conducted in the lithotomy position and under spinal anesthesia. The previously transposed intersphincteric fistula was probed and laid open. This entailed the division of the internal sphincter’s lowest fibers. The tract was curetted, and a small cutback was done to ensure proper drainage and  wound  healing.

### Postoperative care

The cases were moved to the recovery room and then to the internal surgical ward. Analgesia was maintained by IV paracetamol and IV nonsteroidal anti-inflammatory analgesics, if needed. All cases were discharged after the second postoperative day. The patients commenced oral antibiotics (metronidazole 500 mg three times daily and ciprofloxacin 500 mg twice daily for 5 days). Analgesia was achieved by oral paracetamol (1 g/8 h) and oral nonsteroidal anti-inflammatory analgesic. Patients were taught about home wound care.

### Postdischarge recommendations

Diet: encouraging a high-fiber diet. Hygiene: after each bowel movement, soak the anal orifice in a warm sitz bath for 5 min using a moist cotton pad.

### Follow-up

All patients were evaluated twice weekly for 1 week, weekly for 1 month, and monthly for 9 months after the second stage. The time needed for complete wound healing was recorded in all patients. The same policy of postoperative care was followed after every stage of surgery. Postoperative incontinence was evaluated via the Wexner questionnaire after the second procedure. The Cleveland Clinic Fecal Incontinence Severity Scoring System, also known as the Wexner score, is a fecal incontinence score that ranges from 0 to 20, with 0 reflecting ideal continence and 20 representing complete incontinence [7]. That score was calculated for all patients 3, 6, and 9 months after the second stage, keeping in consideration that all patients had a preoperative score of 0, as we already excluded patients who had incontinence during preoperative evaluation. That score was assessed at 3-, 6-, and 9-month follow-up visits.

The recurrence rate was the primary outcome, and the secondary outcomes were incontinence, wound infection, duration until complete wound healing, and operative time.

### Statistical analysis

SPSS v28 (IBM©, Armonk, NY, USA) was employed to conduct the statistical analysis. The normality of the data distribution was assessed using the Shapiro–Wilk test and histograms. The quantitative parametric data were analyzed utilizing an unpaired student *t*-test and presented as the mean and standard deviation (SD). The Mann–Whitney *U* test was employed to analyze quantitative nonparametric data, which were presented as the median and interquartile range (IQR). The chi-squared test or Fisher’s exact test was employed to analyze qualitative variables, which were presented as frequency and percentage (%) when appropriate. The relationship between a dependent variable and one (univariate) or more independent variables (multivariate) was also estimated using logistic regression. Statistical significance was defined as a two-tailed *P*-value that was less than 0.05.

## Results

The mean age of the cases was 43.36 years (range, 18–72 years). Men had a higher prevalence than women, as the former constituted 62.7% of the study participants. Their mean body mass index (BMI) was 29.34 kg/m^2^ (range, 23–37 kg/m^2^). Among the included cases, 84.3% (70 cases) of patients presented with de novo high anal fistulae, while 15.7% (13 cases) had recurrent anal fistulae. Regarding their pre-existing medical comorbidities, hypertension (HTN) and diabetes mellitus (DM) were present in 10.8% and 7.2% cases, respectively. In addition, compensated liver cirrhosis was present in three cases (3.61%). Smokers represented 45.23% of the study population (Table 2).

**Table 2:** Patients’ demographic data, presentation, and disease criteria

		*n* = 83
Age (years)		42.37 ± 13.10
Gender	Male	52 (62.7%)
	Female	31 (37.3%)
BMI (kg/m^2^)		29.34 ± 4.37
Smoking		38 (45.23%)
DM		6 (7.2%)
HTN		9 (10.8)
Compensated liver cirrhosis		3 (3.61%)
Disease duration (months)		7 (3–10)
Patient complaint	Perianal discharge	83 (100%)
	Pain	58 (69.88%)
	Dysdefecation	32 (38.55%)
	Dermatitis	15 (18.07%)
Fistula type	Transsphincteric	74 (89.2%)
	Suprasphincteric	9 (10.8%)
Number of external openings	Single	73(88.0%)
	Multiple	10 (12.0%)
Site of external openings	Anterior	43 (51.81%)
	Posterior	40 (48.19%)
Previous fistula surgery	De novo fistula Recurrent fistula	70 (84.3%) 13 (15.7%)

Data are presented as the mean ± SD, median (IQR), or frequency (%)

*BMI* body mass index, *DM* diabetes mellitus, *HTN* hypertension

The disease duration ranged between 1 and 12 months (median = 7). Perianal discharge was reported in all cases. Other complaints included perianal pain, dysdefecation, and dermatitis, which were reported in 69.88%, 38.55%, and 18.07% of cases, respectively (Table 2).

Most of the detected fistulae were of the transsphincteric type (89.2%), while the remaining cases were of the suprasphincteric type. The majority of patients (88.0%) had a single external opening, whereas the remaining ten cases had multiple openings. Among these, the openings in four cases were close to each other and were included in the tissue core during dissection. The openings in the other six cases were not close to each other; for these six cases, the additional openings were low fistulae and managed with lay open fistulotomy.

The fistula opening location was anterior in 51.81% of cases and posterior in the remaining 48.19% (Table 2).

The operative time of the first stage ranged between 45 and 90 min (mean = 68.13 min), while the hospitalization period ranged between 1 and 2 d (mean = 1.2 d). The duration to complete wound healing had a mean value of 5.49 weeks (range, 4–7 weeks) and delayed healing was encountered in 16 cases (19.28%). Regarding the second stage, the mean operative time was 21.51 min (range, 15–30 min). The duration of hospitalization in the second stage was 1 d. Complete wound healing occurred in 3.1 weeks (range, 2–4 weeks), and delayed healing occurred in 14 cases (16.87%) (Table 3).

**Table 3:** Operative data and hospital stay after the first and second stage (*n* = 83)

	First stage	Second stage
Operative time (min)	68.13 ± 14.5	21.51 ± 4.8
Time to wound healing (weeks)	5.49 ± 1.12	3.1 ± 0.84
Delayed healing	16 (19.28%)	14 (16.87%)
Hospitalization period (d)	1.2 ± 0.3	1.07 ± 0.08

Data are presented as the mean ± SD or frequency (%)

Postoperative infection occurred in only four cases (after the first stage). One of these infections was superficial, and it was managed by frequent dressing, proper hygiene, and IV antibiotics, but the other three cases presented with recurrence later on. Two patients (2.41%) developed tract gangrene that was managed by tract excision and completed staged rerouting later on. No patients developed postoperative bleeding. Recurrence after finishing all stages of the operation occurred in five patients (6.02%). Two patients refused further intervention while the other three patients developed recurrent low transsphincteric fistulae, which were seen in the second stage, and were treated with lay open fistulotomy (Table 4).

**Table 4:** Postoperative complications and recurrence

	*n* = 83
Infection	4 (4.8%)
Gangrene	2 (2.41%)
Bleeding	0 (0%)
Recurrence	5 (6.02%)
Minor incontinence	4 (4.8%)

Data are presented as the frequency (%)

Statistical analysis revealed a significant difference in the Wexner score during follow-up compared with the preoperative value. However, that difference was clinically irrelevant as no patients had a score of more than 4 (Table 5).

**Table 5:** Changes in Wexner score during follow-up (*n* = 83)

Baseline	3 months	6 months	9 months	*P*-value
0 (0–0)	4 (3–4)	3 (2–3)	2 (1–3)	< 0.001*

The bold values and asterisk (*) indicate significant values

Data are presented as the median (IQR). * Significant, as *P*-value ≤ 0.05

The postoperative continence status and Wexner score did not differ from the preoperative continence status and score in 79 patients. Four patients (4.82%) experienced minor postoperative incontinence, in the form of gas incontinence in three patients, and staining of the underwear in one patient.

Univariate analysis for prognostic factors of recurrence revealed that diabetes, suprasphincteric fistula, and infection were independent predictors of recurrence. However, age, gender, BMI, smoking, hypertension, liver disease, duration of disease, the number of fistula openings (single or multiple), the site of the external opening, previous fistula surgery, operative time, and wound healing outcomes were not identified as significant predictors (Table 6). In multivariate regression, suprasphincteric fistula and infection were independent predictors for recurrence (Table 7).

**Table 6:** Univariate regression analysis for prediction of recurrence

Predictors	Univariate regression			
	*P*-value	OR	95% CI for OR	
			Lower	Upper
**Gender**				
Male	R			
Female	0.296	2.679	0.422	17.002
**Age**	0.618	1.018	0.950	1.090
**BMI**	0.626	1.055	0.851	1.307
**Hypertension**	0.507	2.187	0.217	22.042
**Diabetes**	0.026^*^	9.733	1.310	72.319
**Liver disease**	0.999	NA	NA	NA
**Smoking**	0.260	0.277	0.030	2.592
**Fistula type**				
High TR	R			
Suprasphincteric	0.001^*^	58.400	5.543	625.497
**Infection**	< 0.001^*^	115.500	8.053	1656.492
**Bleeding**	1.000	NA	NA	NA
**Disease duration**	0.097	0.766	0.558	1.050
**Site of external openings**				
Anterior	0.707	1.425	0.226	9.004
Posterior	R			
**Number of external openings**				
Single	R			
Multiple	0.074	5.833	0.844	40.307
**Previous fistula surgery**				
De novo fistula	R			
Recurrent fistula	0.999	NA	NA	NA
First stage operative time	0.539	1.020	0.957	1.088
First stage wound healing	0.318	0.653	0.284	1.505
Second stage operative time	0.471	0.929	0.759	1.136
Second stage wound healing	0.266	0.585	0.227	1.505

The bold values and asterisk (*) indicate significant values

*OR* odds ratio, *CI* confidence interval, *LL* lower limit, *UL* upper limit

**Table 7:** Multivariate regression analysis for prediction of recurrence

Predictors	Multivariate regression			
	*P*-value	OR	95% CI for OR	
			Lower	Upper
Diabetes mellitus	0.122	13.481	0.497	365.632
Suprasphincteric type	0.032^*^	23.355	1.305	418.115
Infection	0.032^*^	55.547	1.401	2203.014

The bold values and asterisk (*) indicate significant values

*OR* odds ratio, *CI* confidence interval, *LL* lower limit, *UL* upper limit

## Discussion

A fistula is referred to as an unusual communication between two epithelialized surfaces. Anal fistulae are characterized by an atypical communication between the anorectal canal and the perianal epidermis [8].

Laying open of the fistulous tract is the classic operation for anal fistula management and is associated with minimal recurrence [9, 10]. The low recurrence rate after fistulotomy is probably because of the internal opening elimination [11]. This allows the fistulous tract to heal from the inside out. In addition, by opening the tract, any infection or abscess can drain freely, reducing the risk of recurrence, a benefit not typically seen in sphincter-preserving surgeries. In these procedures, the internal opening is merely blocked if fibrin glue or a fistula plug is used, or is covered in a mucosal advancement flap operation, stitched in video-assisted anal fistula treatment and ligation of the intersphincteric fistula tract techniques, or burned in operations using laser technology. The reported rate of recurrence after anal fistula surgery is between 3% and 57%, with varying rates among different procedures [12]. It is thus not astonishing that the recurrence rate is higher after sphincter-saving fistula surgery as compared with fistulotomy [13, 14]. Despite the low recurrence rate after fistulotomy, this operation’s major disadvantage is the inevitable division of part of the anal sphincters, which can lead to postoperative fecal incontinence [9, 15–17]. It thus seems that recurrence and incontinence are two faces of the same coin that accompany surgery for anal fistula; the more that is done to avoid one, the more it is likely to get the other [17].

Fistula tract rerouting is a minimally sphincter-sacrificing procedure in which the extrasphincteric portion of the tract is transposed into an intersphincteric position. Fistulotomy of the transposed intersphincteric tract is then performed at a later stage [5]. Mann and Clifton were the first to describe that method [5].

This study is the first to discuss this technique with such a large number of cases (83 cases) and also analyzes the recurrence causes.

In the current study, most of the detected fistulae were of the transsphincteric type (89%) while the remaining cases were suprasphincteric. In agreement with our results, Abou-Zeid et al. [18] stated that transsphincteric fistula was the most common type (68.5%), while the remaining cases were the suprasphincteric type (31.5%). In addition, Omar and his colleagues reported that transsphincteric fistula was the most common type (93.33%). Other types included horseshoe and suprasphincteric fistulae (3.33% for each) [19].

Our findings revealed no incidence of postoperative bleeding in our patients.

Postoperative infection occurred in only four cases (5%); one of these cases was superficial and managed by repeated dressing, proper hygiene, and intravenous antibiotics, but the other three cases developed recurrence. In a previous similar study, the incidence of the same complication was 5% after the rerouting procedure [20], which is similar to our findings.

In the current study, two patients developed postoperative tract gangrene. Gangrene occurs because the mobilized fistulous tract was thinned out extensively to allow it to pass through the small slit in the external sphincter before it was transposed to the intersphincteric space. This probably jeopardized the vascularity of the tract which became gangrenous in its distal part. Abou-Zeid et al. [18] reported that gangrene of the mobilized rerouted tract occurred in one patient (1.85%).

Our findings revealed that the healing period after the first stage ranged between 4 and 7 weeks (28–42 d), and the healing period after the second stage was between 2–4 weeks (14–28 d). In the study conducted by Ouf et al., the healing period had a mean value of 43.4 d (range, 35–53 d). The authors did not specify whether it is the period needed for a specific stage or all stages. In addition, they did not mention a specific definition of complete healing [20]. Differences in the healing rate between studies could be explained by patient factors and the incidence of postoperative complications.

Our finding revealed that the postoperative continence status and Wexner score did not differ from the preoperative continence status and score in 79 patients. Four patients (4.82%) experienced minor postoperative incontinence, in the form of gas incontinence in three patients and staining of the underwear in one patient. Two patients improved after training their pelvic floor muscles with regular exercises, and the other two did not improve through the follow-up period. However, that difference was clinically irrelevant, as no patients had a score of more than 4. Likewise, Abou Zaid et al. [18] reported that the postoperative continence status and Wexner score did not differ from the preoperative continence status and score in their enrolled 54 patients.

In the same context, Maqsood and Rasikh [21] reported the incidence of flatus incontinence in only one patient after rerouting for high fistulae (2.7%), while Ouf et al. [20] showed no incidence of that complication after the same procedure (0%).

Other authors reported a relatively higher incidence of the same adverse event. For instance, Ibrahim et al. reported that postoperative incontinence occurred in only 10% of cases after the rerouting procedure (three cases). Two of them had only gas incontinence while the remaining case had stool incontinence [22].

In our study, postoperative recurrence was encountered in 6% of cases. In accordance with our findings, Ouf and his coworkers [20] reported that recurrence was encountered in 10% of patients who had the same procedure for high perianal fistula, which is similar to our findings.

In contrast, other studies reported lower recurrence rates after the same intervention in such cases. According to the study of Ibrahim et al., [22] recurrence occurred in only two cases after the same procedure (6.7%).

Univariate analysis for prognostic factors of recurrence revealed that diabetes, suprasphincteric fistula, and infection were independent predictors of recurrence. However, age, gender, BMI, smoking, hypertension, liver disease, duration of disease, the number of fistula openings (single or multiple), the site of the external opening, previous fistula surgery, operative time, and wound healing outcomes were not identified as significant predictors. In multivariate regression, suprasphincteric fistula and infection were independent predictors.

Mei and colleagues identified factors such as prior anal surgery, high transsphincteric fistula, undetected internal openings, and multiple fistulous tracts as being associated with an increased risk of recurrence [12]. However, these factors did not align with the findings of our study. Jordan et al. concluded that suprasphincteric fistulae are the greatest risk factors for recurrence and incontinence, making them the most challenging to treat [11].

In our study, we assessed five cases of fistula recurrence. Among these, one was a high transsphincteric fistula, while the other four were suprasphincteric. Notably, three of the suprasphincteric fistulae developed postoperative infections following the first stage of surgery. Suprasphincteric fistulae require particularly careful and precise dissection. We hypothesize that minor, unnoticed punctures occurred during the dissection of the tract, leading to the spread of infection. This likely happened through the external sphincter opening, which had been cored during the procedure. The infection, originating from these small punctures, contributed to the formation of recurrent fistulae. These issues were observed early in the study, during the learning curve phase. In subsequent cases, we modified our approach by ensuring a more careful dissection of the tract within a core of healthy tissue. This technique was effective in preventing tract gangrene and small punctures, and thereby reducing the risk of infection and recurrence.

We recommend that more studies involving more cases from different surgical centers should be performed in the future; these studies should assess long-term follow-up.

**Limitations**: Our study lacks intermediate and long-term follow-up, as well as only studying a small sample size of patients with suprasphincteric fistulae.

## Conclusions

The rerouting procedure is a feasible and safe surgical option for managing high transsphincteric perianal fistulae. It is associated with low postoperative complication rates, including short-term recurrence. It combines the advantages of fistulotomy and sphincter-preserving fistula surgery. However, further studies involving a larger number of suprasphincteric fistula cases are needed to evaluate the efficacy of the rerouting technique in treating such fistulae.

## Data Availability

No datasets were generated or analyzed during the current study.
